# Supplementary figures and images for: The selenoprotein P 3’ untranslated region is an RNA binding protein platform that fine tunes selenocysteine incorporation
Source: PLoS One. 2022 Jul 29;17(7):e0271453. doi: 10.1371/journal.pone.0271453 (PMC9337670; doi:10.1371/journal.pone.0271453)

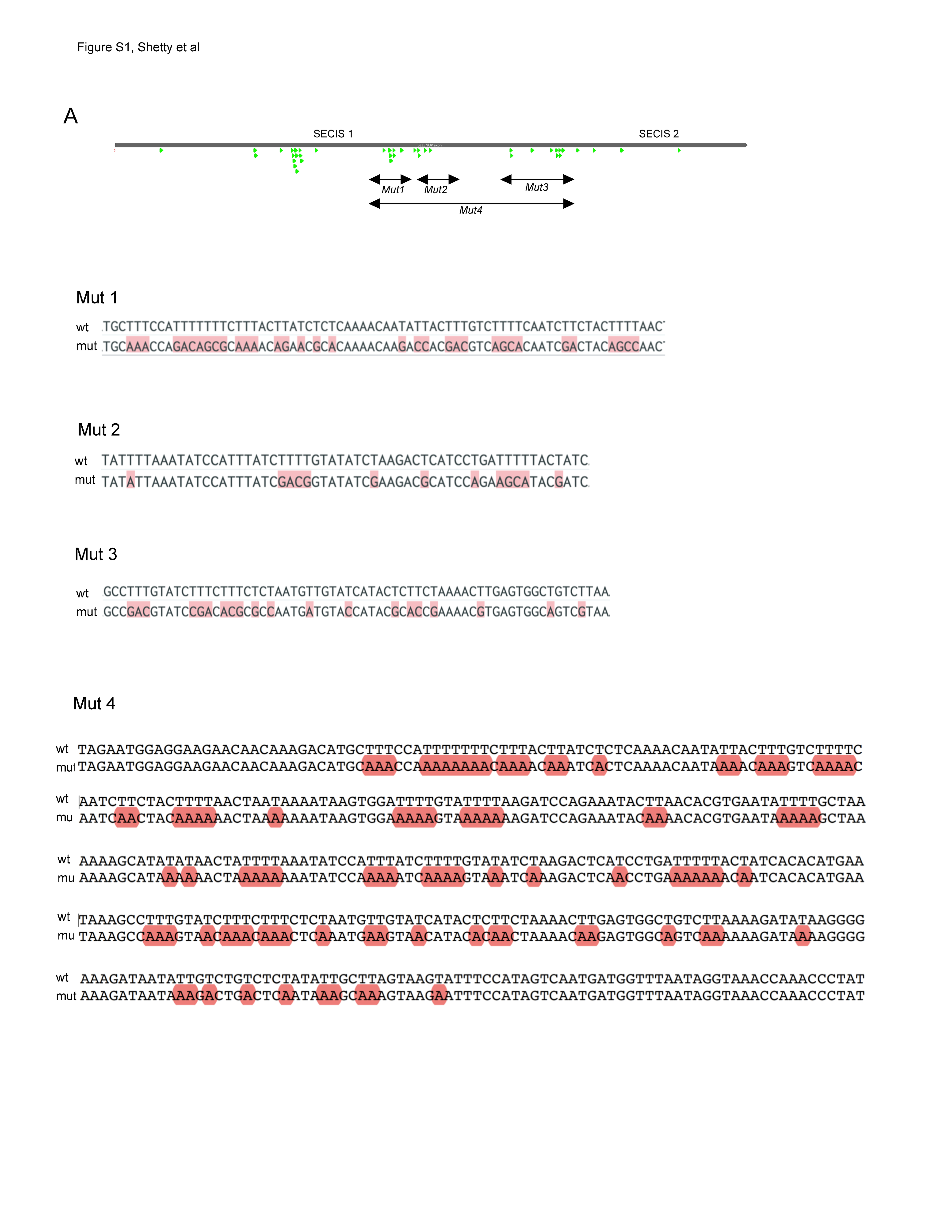

Supplement: S1 Fig — (TIF) [file pone.0271453.s001.tif]

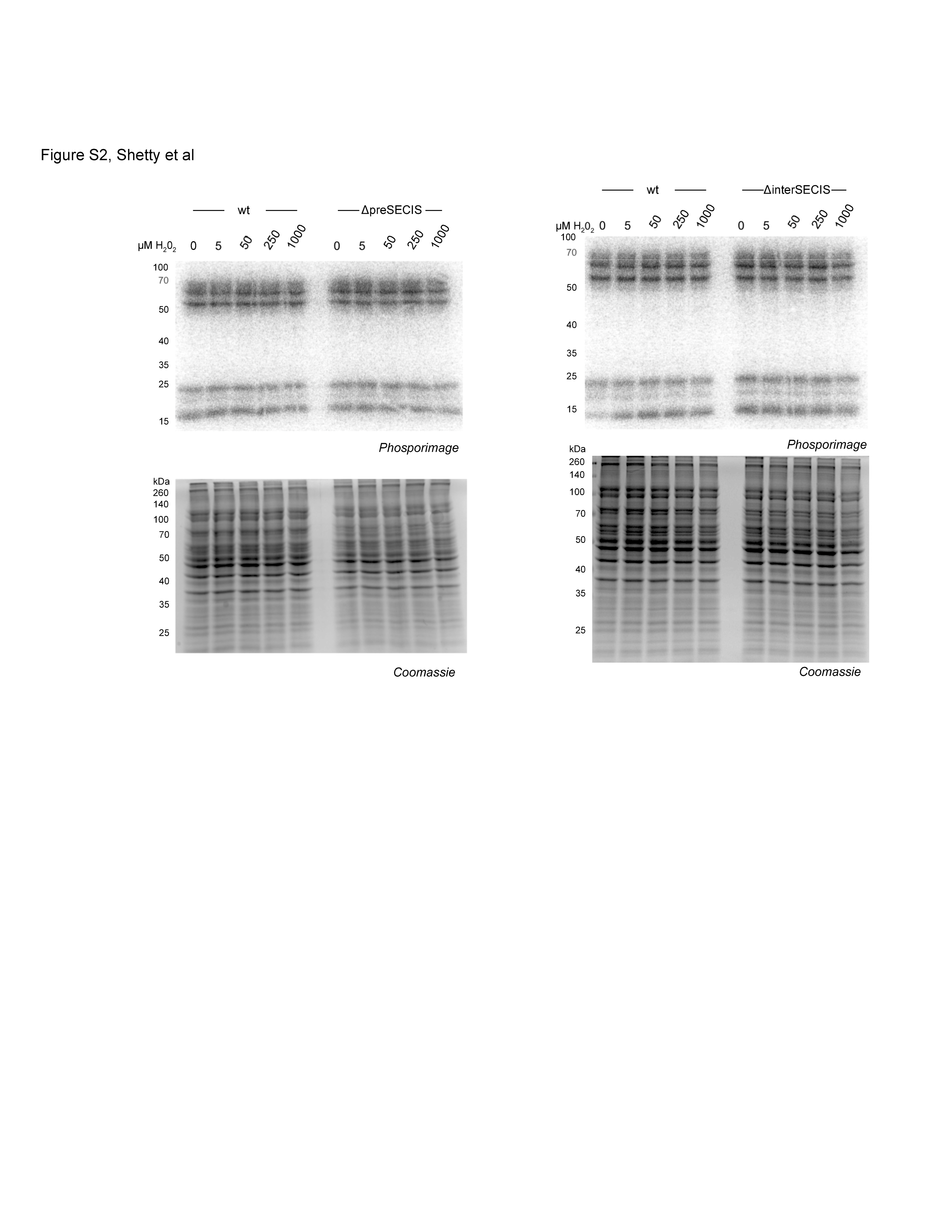

Supplement: S2 Fig — Cell lysates derived from the 75Se-selenite labeling described for Fig 5 were analyzed by SDS-PAGE followed by phosphorimage analysis. (TIF) [file pone.0271453.s002.tif]
