# Supplementary material for: The selenoprotein P 3’ untranslated region is an RNA binding protein platform that fine tunes selenocysteine incorporation
Source: PLoS One. 2022 Jul 29;17(7):e0271453. doi: 10.1371/journal.pone.0271453 (PMC9337670; doi:10.1371/journal.pone.0271453)
Supplement: S1 Raw images — The numbers below gels correspond to loading order shown in figures. (PDF) [file pone.0271453.s003.pdf]

Figure 2B

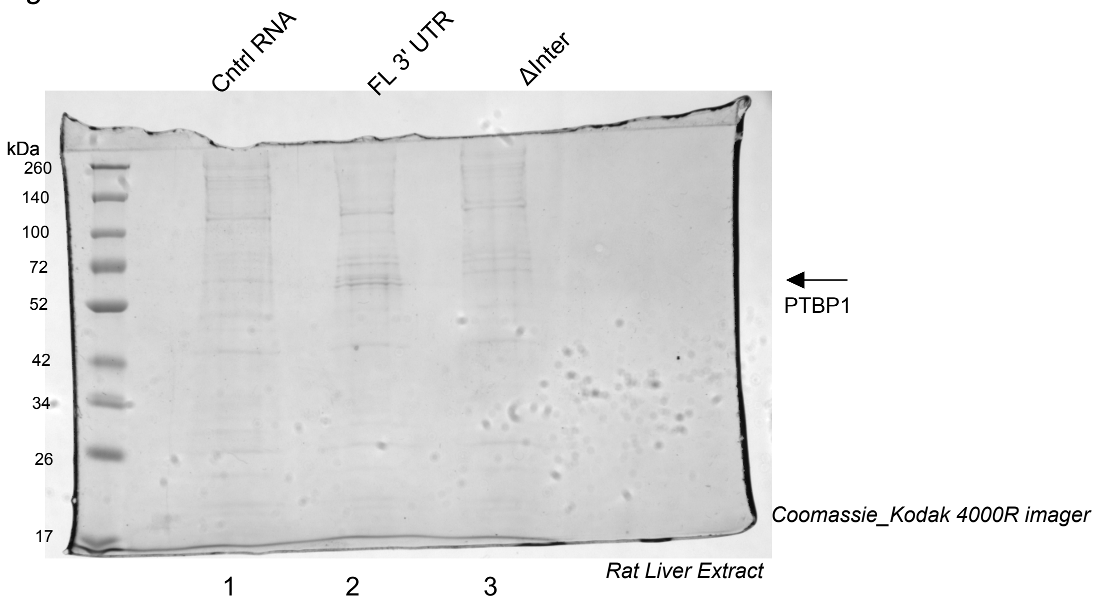

Figure 2C

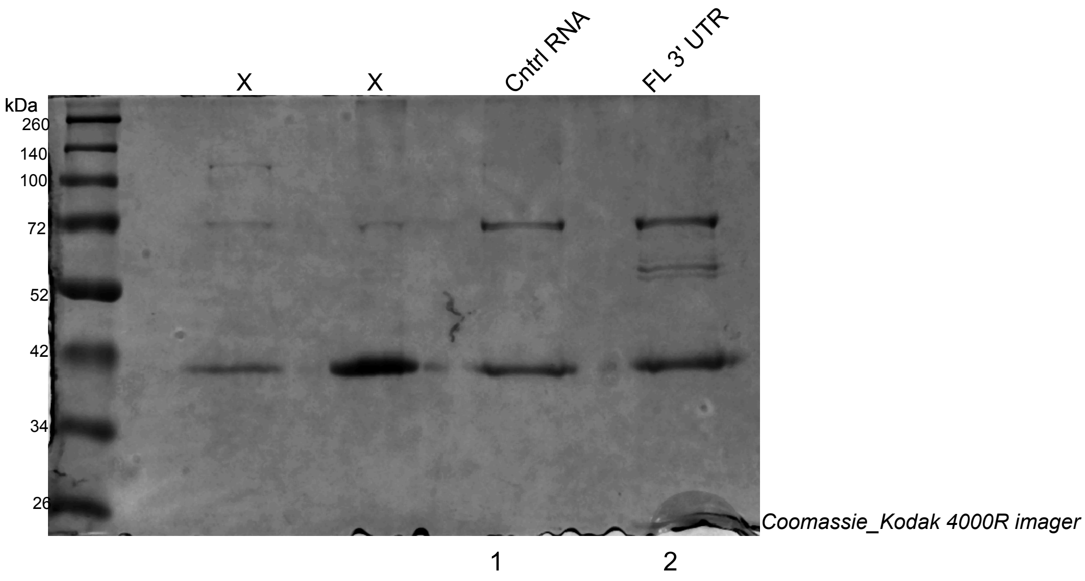

Figure 2D

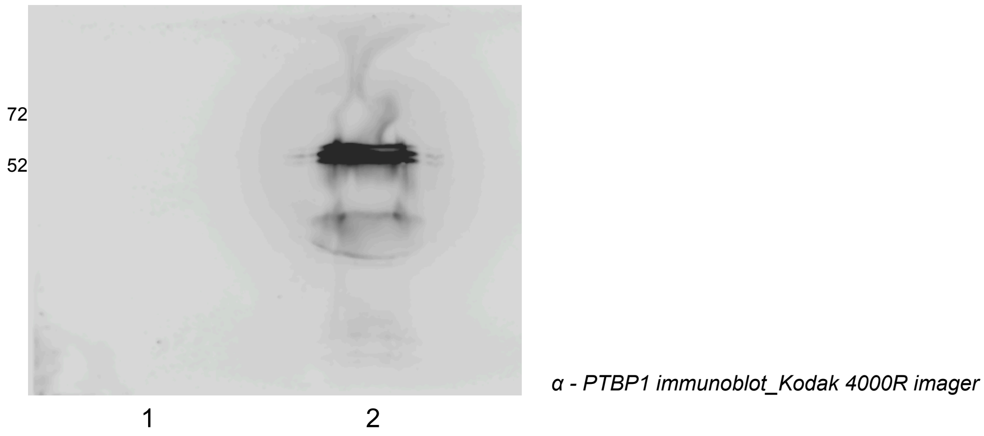

Figure 3B

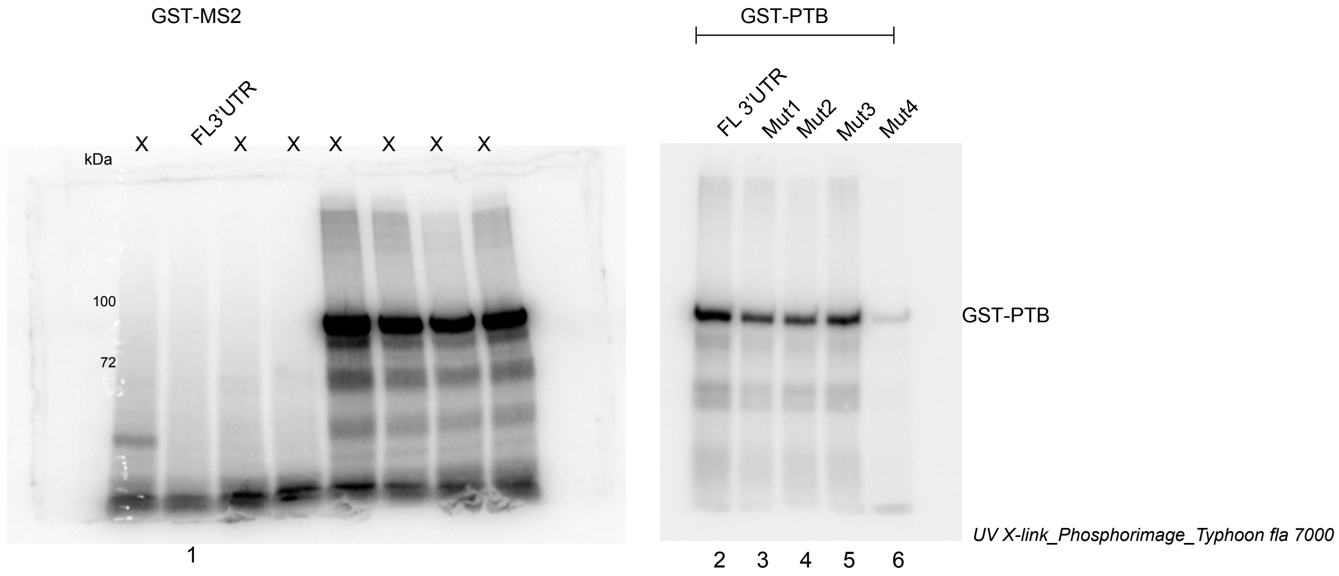

GST-MS2
GST-PTB
X
FL3'UTR
X
X
X
X
X
X
kDa
100
72
FL 3'UTR
Mut1
Mut2
Mut3
Mut4
GST-PTB
UV X-link_Phosphorimage_Typhoon fla 7000
1
2 3 4 5 6

Figure 4B

bp △Pre △Inter wt X X X X X X

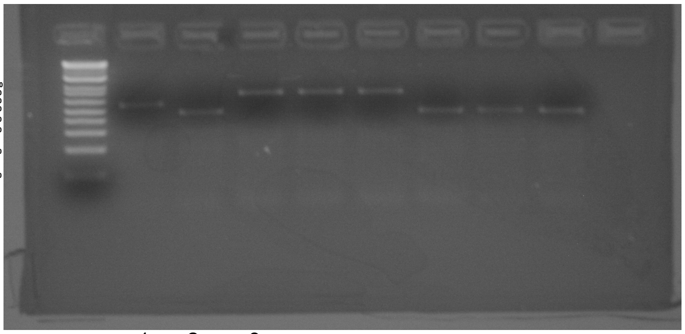

gDNA_EOS Digital Rebel XSi

1 2 3

RT △Pre + △Inter + X wt + △Pre - △Inter - X wt -

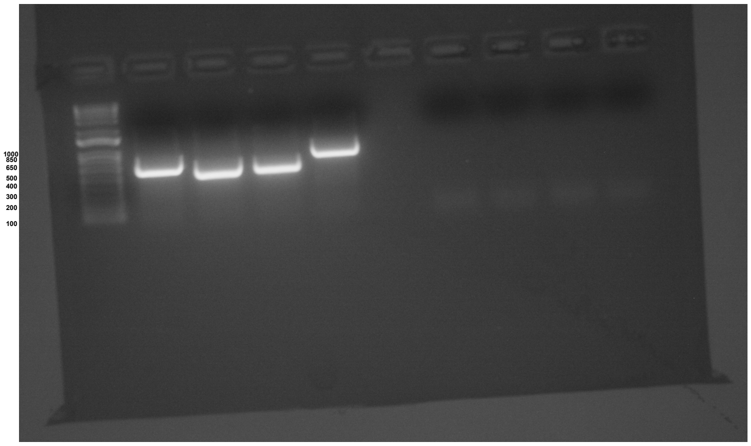

cDNA_EOS Digital Rebel XSi

1 2 3 4 5 6

Figure 4C X X X X X X X

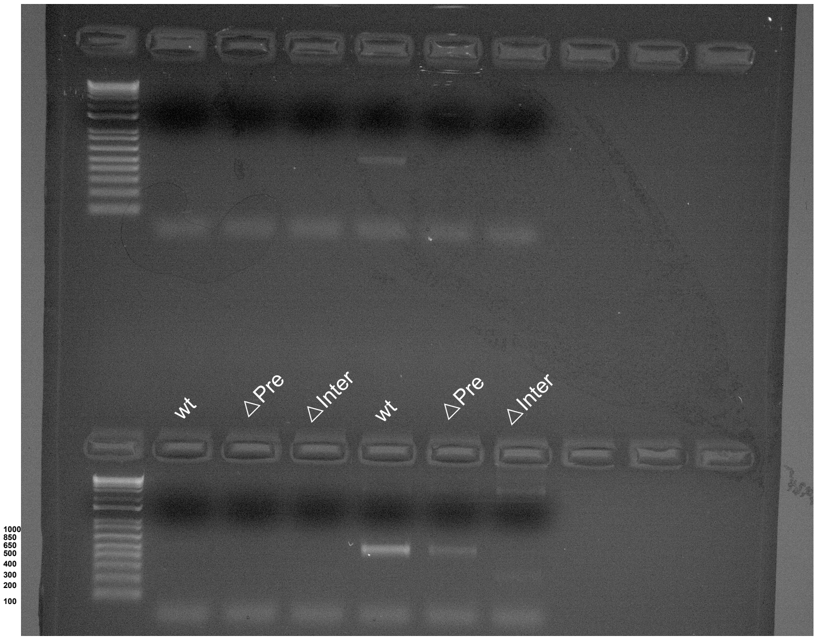

EOS Digital Rebel XSi

α–FLAG IP α–PTBP1 IP

1 2 3 4 5 6

Figure 5A

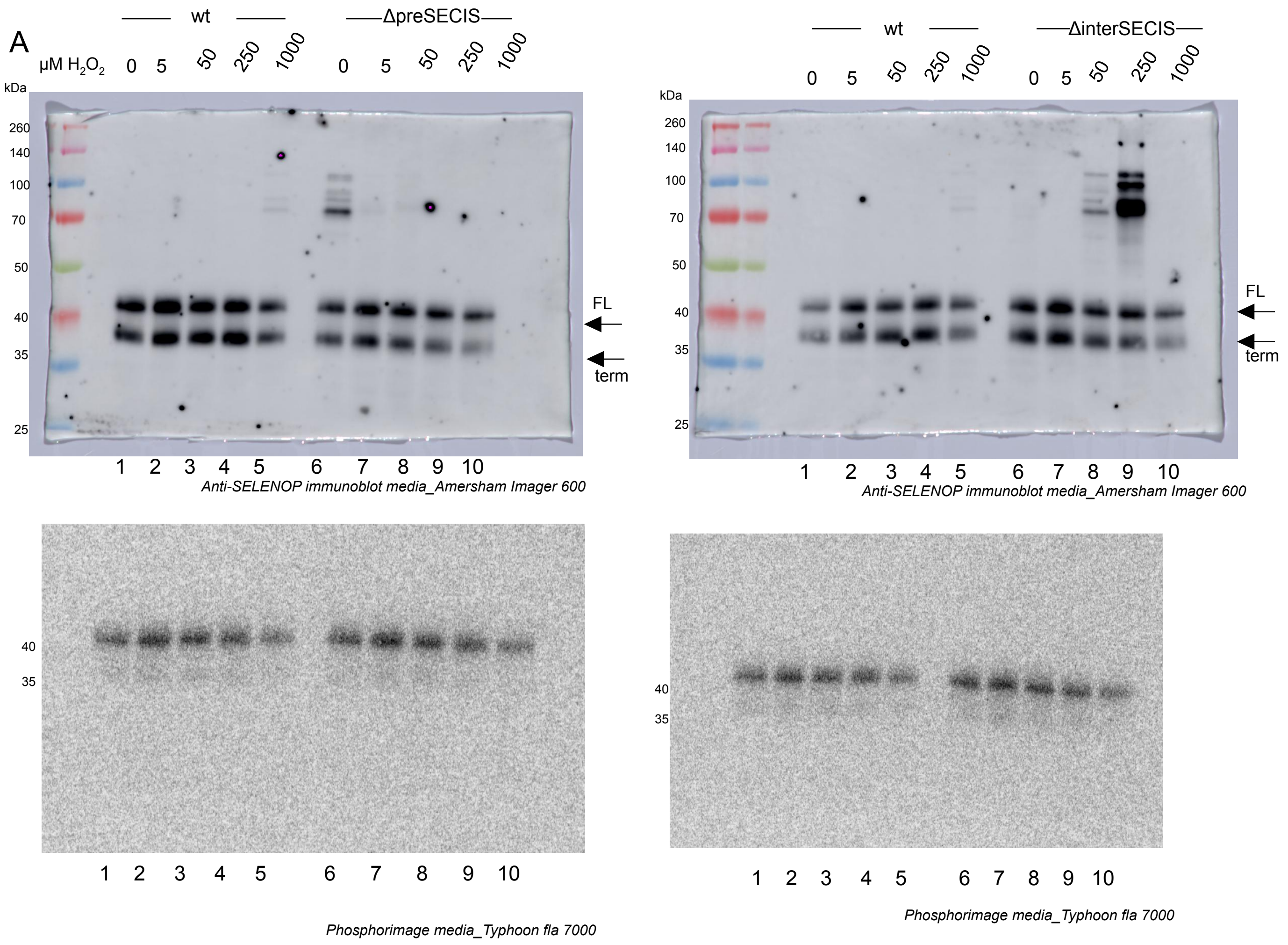
A
µM H2O2
wt
ΔpreSECIS
0 5 50 250 1000 0 5 50 250 1000
kDa
260
140
100
70
50
40
35
25
FL
term
1 2 3 4 5 6 7 8 9 10
Anti-SELENOP immunoblot media_Amersham Imager 600
wt
ΔinterSECIS
0 5 50 250 1000 0 5 50 250 1000
kDa
260
140
100
70
50
40
35
25
FL
term
1 2 3 4 5 6 7 8 9 10
Anti-SELENOP immunoblot media_Amersham Imager 600
40
35
1 2 3 4 5 6 7 8 9 10
Phosphorimage media_Typhoon fla 7000
40
35
1 2 3 4 5 6 7 8 9 10
Phosphorimage media_Typhoon fla 7000

Figure 6A

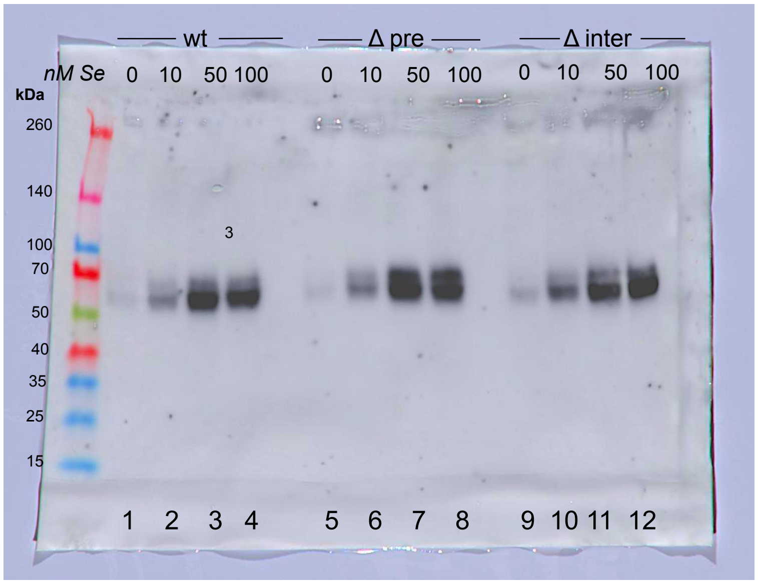

Immunoblot Media_Amersham Imager600

Figure 6B

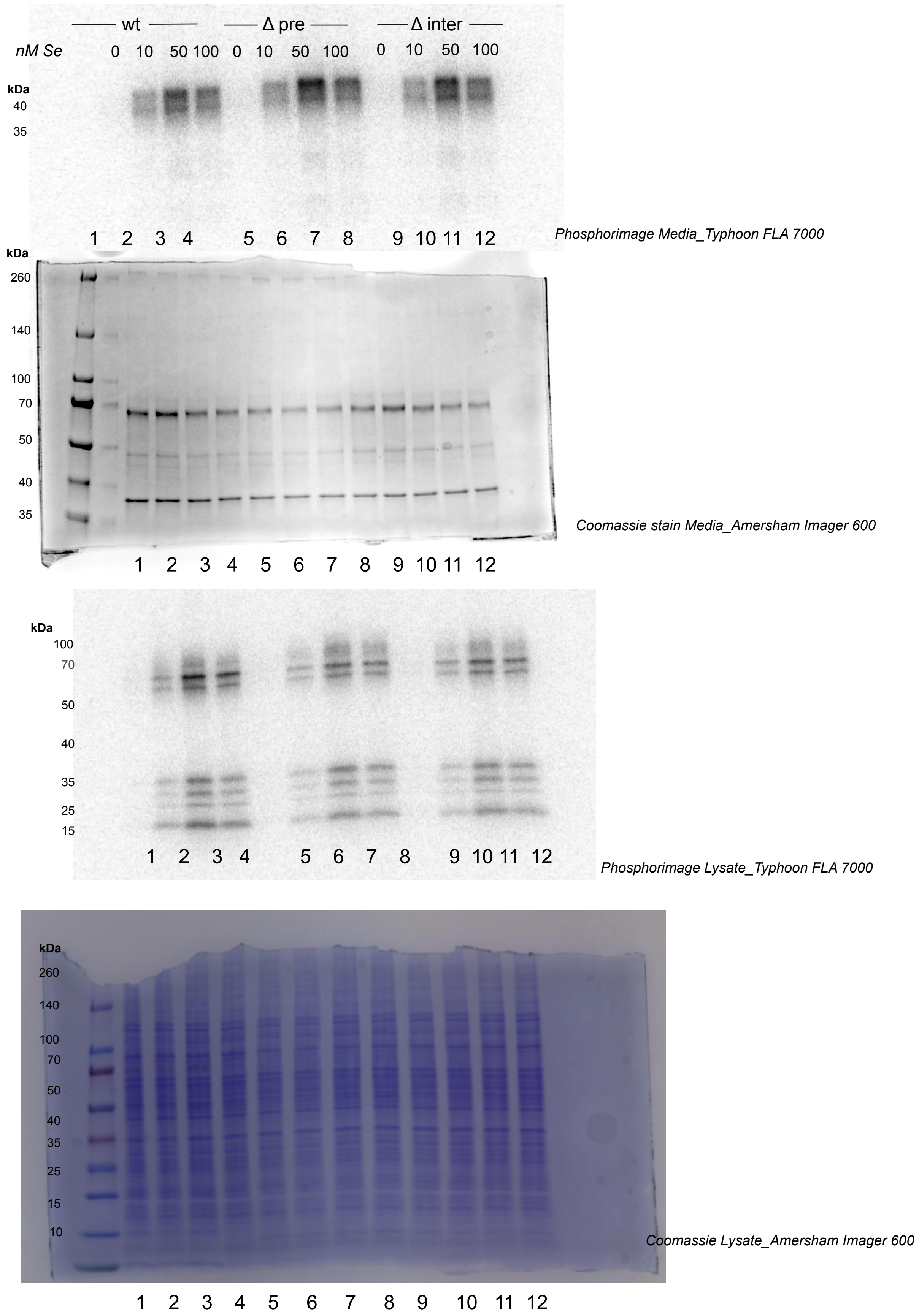
wt
Δ pre
Δ inter
nM Se
0 10 50 100 0 10 50 100 0 10 50 100
kDa
40
35
1 2 3 4 5 6 7 8 9 10 11 12
Phosphorimage Media_Typhoon FLA 7000
kDa
260
140
100
70
50
40
35
1 2 3 4 5 6 7 8 9 10 11 12
Coomassie stain Media_Amersham Imager 600
kDa
100
70
50
40
35
25
15
1 2 3 4 5 6 7 8 9 10 11 12
Phosphorimage Lysate_Typhoon FLA 7000
kDa
260
140
100
70
50
40
35
25
15
10
Coomassie Lysate_Amersham Imager 600
1 2 3 4 5 6 7 8 9 10 11 12

Supplementary Figure S2

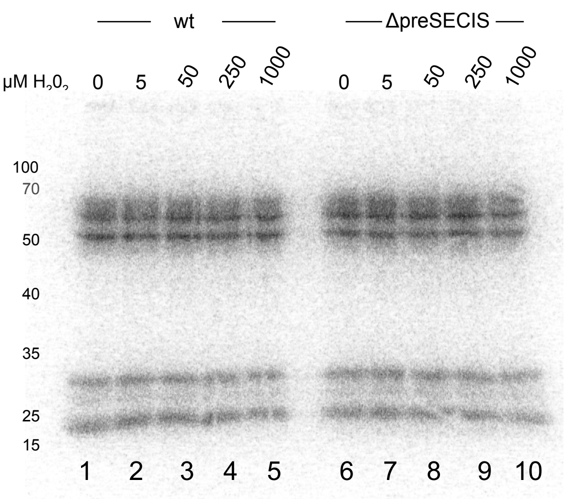

Phosporimage_Amersham Imager 600

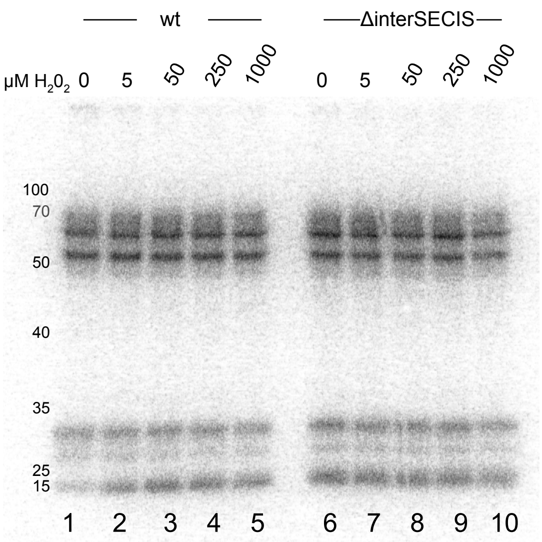

Phosphorimage_Amersham Imager 600

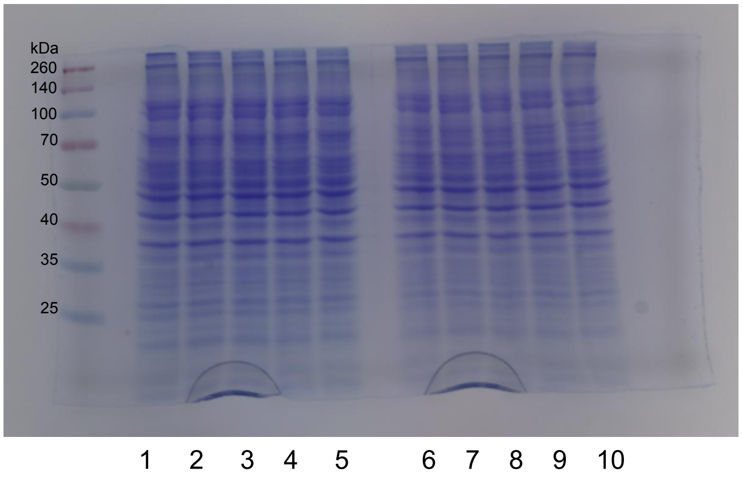

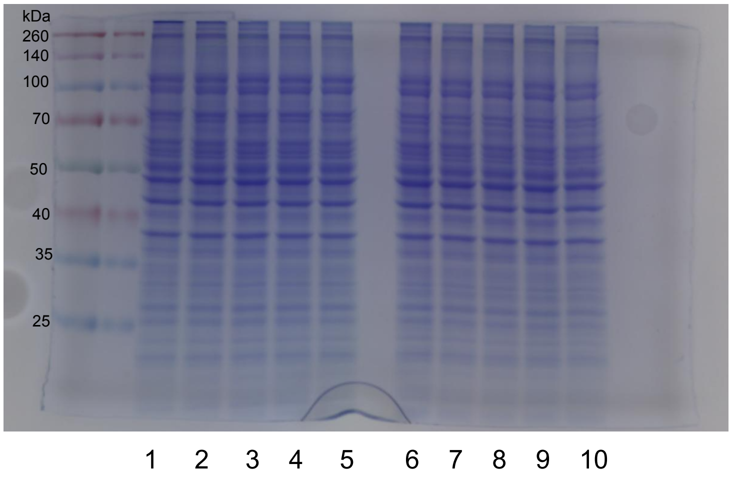

Coomassie_Amersham Imager600

Coomassie_Amersham Imager600
